# Characterization of *Rolled and Erect Leaf 1* in regulating leave morphology in rice

**DOI:** 10.1093/jxb/erv319

**Published:** 2015-07-02

**Authors:** Qiaoling Chen, Qingjun Xie, Ju Gao, Wenyi Wang, Bo Sun, Bohan Liu, Haitao Zhu, Haifeng Peng, Haibing Zhao, Changhong Liu, Jiang Wang, Jingliu Zhang, Guiquan Zhang, Zemin Zhang

**Affiliations:** ^1^State Key Laboratory for Conservation and Utilization of Subtropical Agro-Bioresources, Guangdong Provincial Key Laboratory of Plant Molecular Breeding, South China Agricultural University, Guangzhou, 510642, China; ^2^Shanghai Institute of Plant Physiology and Ecology, Chinese Academy of Sciences, Shanghai, 20032, China

**Keywords:** BiFC, BR, erect leaf, rice, rolled leaf, yeast two hybrid.

## Abstract

Leaf morphology, particularly in crop, is one of the most important agronomic traits because it influences the yield through the manipulation of photosynthetic capacity and transpiration. To understand the regulatory mechanism of leaf morphogenesis, an *Oryza sativa* dominant mutant, *rolled and erect leaf 1* (*rel1*) has been characterized. This mutant has a predominant rolled leaf, increased leaf angle, and reduced plant height phenotype that results in a reduction in grain yield. Electron microscope observations indicated that the leaf incurvations of *rel1* dominant mutants result from the alteration of the size and number of bulliform cells. Molecular cloning revealed that the *rel1* dominant mutant phenotype is caused by the activation of the *REL1* gene, which encodes a novel unknown protein, despite its high degree of conservation among monocot plants. Moreover, the downregulation of the *REL1* gene in the *rel1* dominant mutant restored the phenotype of this dominant mutant. Alternatively, overexpression of *REL1* in wild-type plants induced a phenotype similar to that of the dominant *rel1* mutant, indicating that *REL1* plays a positive role in leaf rolling and bending. Consistent with the observed *rel1* phenotype, the *REL1* gene was predominantly expressed in the meristem of various tissues during plant growth and development. Nevertheless, the responsiveness of both *rel1* dominant mutants and *REL1*-overexpressing plants to exogenous brassinosteroid (BR) was reduced. Moreover, transcript levels of BR response genes in the *rel1* dominant mutants and *REL1*-overexpressing lines were significantly altered. Additionally, seven REL1-interacting proteins were also identified from a yeast two-hybrid screen. Taken together, these findings suggest that *REL1* regulates leaf morphology, particularly in leaf rolling and bending, through the coordination of BR signalling transduction.

## Highlight

The *Rolled and Erect Leaf 1* (*REL1*) gene is a novel component controlling brassinosteroid signalling-associated leaf morphogenesis and leaf angle in *Oryza sativa*.

## Introduction

In plant, leaf development is a complex process comprising cell division and expansion, axis determination, and tissue differentiation and specification (Moulia, 2000). In terms of *Oryza sativa* (rice), the leaf is polarized along the adaxial–abaxial axis (Itoh *et al.*, 2005). Moderate leaf rolling can maximize the rice yield through more efficient photosynthesis and reduced transpiration (Lang *et al.*, 2004; Zhang *et al.*, 2009; Zou *et al.*, 2011), as well as by increasing stomatal resistance, reducing water loss, and the resulting erection of the leaf blade (King *et al.*, 1996; Moulia, 2000; Sakamoto *et al.*, 2006). Therefore, how to increase crop productivity and yield through manipulating the adaxial and abaxial cells has become an important issue in agriculture (Zou *et al.*, 2011).

Advances in research suggest that alternation of bulliform cells arranged on the adaxial epidermis of the leaf leads to adaxial or abaxial rolling of mature leaves (Botwright *et al.*, 2005; Fujino *et al.*, 2008). Recent studies demonstrated that hypodermis cells were involved in leaf rolling in higher plants as well (Kadioglu and Terzi, 2007). To date, more than 10 rice leaf rolling–associated genes have been reported to be involved in abaxial and adaxial polarity establishment (Zou *et al.*, 2011). For example, mutation of *ROLLED LEAF 9* (*RL9*)/*SHALLOT-LIKE1* caused an extreme rolled leaf phenotype by impairing programmed cell death of abaxial mesophyll cells (Yan *et al.*, 2008; Zhang *et al.*, 2009). Notably, most of the leaf rolling mutants exhibited an abaxially rolled leaf phenotype, such as *abaxially curled leaf 1*(*acl1*) and *acl2* (Li *et al.*, 2010). However, there are some regulators involved in regulation of the adaxially rolled leaf, such as *ADAXIALIZED LEAF1* and *Rice outermost cell-specific gene 5* (Hibara *et al.*, 2009; Zou *et al.*, 2011). In addition, other regulators have also been well characterized. For example, *Narrow and Rolled Leaves 1* participates in regulating leaf morphology through coordinating the regulation of *CONSTITUTIVELY WILTED1*/ *NARROW LEAF7*, *RL9*, and *OsAGO7* (Wu *et al.*, 2010).

Another feature of leaf morphology is the leaf bending at the lamina joint, which results from the unequal elongation that occurs between the adaxial and abaxial cells (Itoh *et al.*, 2005). Accumulating evidence has illustrated that brassinosteroids (BRs) play a pivotal role in leaf bending (Bishop and Yokota, 2001; Hong *et al.*, 2004; Sakamoto *et al.*, 2006; Tanaka *et al.*, 2009; Tong *et al.*, 2009; Tong *et al.*, 2012; Zhang *et al.*, 2012). For example, depleted rice BR receptor (*OsBRI1*) mutants exhibit a predominant erect leaf phenotype (Yamamuro *et al.*, 2000). Similarly, suppression of *OsBZR1* results in the erect leaf phenotype (Hong *et al.*, 2004; Sakamoto *et al.*, 2006; Bai *et al.*, 2007). Intriguingly, *LEAF and TILLER ANGLE INCREASED CONTROLLER* (*LIC*) regulate leaf bending through inhibition of the transcription of *OsBZR1*, by binding to its promoter (Zhang *et al.*, 2012). *Dwarf and Low-Tillering* (*DLT*) is another newly identified gene participating in leaf morphology. Tong and associates showed that *OsBZR1* mediated leaf morphology through the repression of *DLT* by binding BR-response element in its promoter (Tong *et al.*, 2009). Further research indicated that *OsBIN2* also participated in establishment of leaf morphology through the interaction with *DLT* (Tong *et al.*, 2012). However, the regulatory mechanism of BR-mediated leaf morphology in rice still needs to be further elucidated.

Here, a novel gene, *REL1*, has been characterized. *REL1* encodes an unknown protein and plays a positive role in leaf rolling and bending. Detailed analyses indicated that leaf rolling of *rel1* mutants was caused by the altered profile of bulliform cells. The results also suggest that *REL1* may regulate leaf bending by coordinated expression of BR-associated genes. In addition, seven REL1-interacting proteins were identified through a yeast two-hybrid screen.

## Materials and methods

### Plant materials and growth conditions

Rice cultivar ‘Zhonghua 11’ was used as the wild type. All rice seeds in this study were propagated in the paddy field in Guangzhou, China. For laboratory work, rice plants were grown in a greenhouse under a 16-h-light/8-h-dark cycle at 30°C. No significant differences were observed when *rel1* mutants were grown in the greenhouse compared to the paddy field.

### Electron microscopy

The young leaf was prepared as a semithin cross-section, and the samples were fixed by 2% (v/v) OsO_4_ in phosphate buffer, air-pumped for 4–48h, and then transferred to 4°C overnight. The fixed solution was then discarded, and the samples were washed three times with 0.1M phosphate buffer (pH 7.3). Samples were dehydrated in a graded ethanol series and then submerged in glycomethacrylate solution (GMA to 95% ethanol at 1:1) at 4°C overnight, and then transferred to pure GMA at room temperature for 2–3h or 4°C overnight. Finally, the samples were embedded and the corresponding strips spliced for electron microscopy (HITACHI S-3000N electron microscope) and photographed. A paraffin cross-section assay was performed as previously described (Li *et al.*, 2010) with minor modifications. Briefly, 2-week-old leaf samples were fixed with 50% formalin–acetic acid–alcohol solution and then stored at 4°C overnight. Subsequently, the samples were dehydrated in a graded ethanol series and the ethanol then substituted with a graded isoamyl acetate series. Afterwards, the samples were transferred to xylene and then embedded in paraffin. Strips of paraffin slices (2–3 μm) were spread at 42°C on a hot platform overnight. The slices were stained using 1% Toluidine Blue O at 37°C for 5min, and dried at 37°C after washing with water. After removal of paraffin using xylene, the slices were sealed for observation under the microscope (HITACHI S-3000N electron microscope) and photos captured.

### Analysis of the T-DNA insertion locus in *rel1* mutant

Thermal asymmetric interlaced polymerase chain reaction (PCR) was used to isolate the flanking sequence of T-DNA (Liu and Whittier, 1995). Nested primers of the T-DNA right border and the degenerated primers were TR1, TR2, TR3, and AD2-4. Primers for testing of the T-DNA inserting locus were rel1-39208 and TDNA7777 for the right site and rel1-72833 and TDNA10622 for the left. Sequences of the primers are supplied in Supplementary Table S1.

### Plasmid construction and rice transformation

To generate the *REL1* antisense construct, a 294bp fragment from the *REL1* coding region to the 3′-untranslated region was amplified from rice cultivar ‘Zhonghua 11’ (wild type) cDNA templates using their corresponding primer pairs listed in Supplementary Table S1, and then the fragment was cloned into the binary vector pCAMBIA1301-Ubinos. To produce the *REL1* overexpression transgenic plants, the full-length *REL1* coding sequences was amplified from cDNA derived from rice ‘Zhonghua 11’ using their corresponding primer pairs listed in the Supplementary Table S1. After confirmation by DNA sequencing, each amplified sequence was cloned into the binary vector pCAMBIA1301-Ubinos for antisense or overexpression of *REL1*. The final constructs were electroporated into *Agrobacterium tumefaciens* strain EHA105 for rice.

In respect to the GUS assay, a 2545bp DNA fragment immediately upstream of the start codon of *REL1* was amplified by using the corresponding specific primers (see Supplemental Table S1). The resulting REL1:GUS reporter gene was introduced into the wild-type plant by *A. tumefaciens*-mediated transformation. Transgenic plants were selected on Hygromycin medium and T3 transgenic plants were used to analyse GUS activity. To generate the REL1-green fluorescent protein (GFP) construct to examine subcellular localization, the pCAMBIA1300-35S-REL1-GFP construct was produced by the corresponding primers listed in Supplementary Table S1.

### Histological GUS assay

The GUS activity analysis was made by using a standard protocol (Jefferson *et al.*, 1987). Briefly, transgenic plant tissues were incubated in X-Gluc buffer (0.1mol L^−1^ K_2_HPO_4_ (pH 7.0), 0.1mol L^−1^ KH_2_PO_4_ (pH 7.0), 5 mmol L^−1^ K_3_Fe (CN)_6_, 5 mmol L^−1^ K_4_Fe(CN)_6_•3H_2_O, 0.1% Triton X-100, 20% methanol, 1mg mL^−1^ X-Gluc) at 37°C for 2 hours. The samples were cleared of chlorophyll by dehydration with ethanol. The stained samples were photographed using a Cannon digital camera and stereoscope (OLYMPUS SZX12).The stained stamen was sliced using resin sections (Leica Historesin) and analysed by light microscopy (OLYMPUS BX51).

### BR response assay

The lamina joint assay using the micro-drop method was performed as described previously (Tong *et al.*, 2009) with minor modifications. Briefly, 100nM L^−1^, 10nM L^−1^, and 1 µM L^−1^ of 24-epibrassinolide (epiBL) dissolved in ethanol was respectively spotted onto the top of lamina after 10 days’ germination and 3 days’ growth at 30°C. Images were taken after 3 days’ incubation, and the angles of lamina joint bending were measured.

### RNA extraction and quantitative real-time PCR

Total RNA was extracted using the RNeasy Plant Mini Kit (Qiagen) according to the manufacturer’s instructions. The first strand of cDNA was synthesized using TransScript First-Strand cDNA Synthesis SuperMix (TransGen Biotech) and quantitative real-time polymerase chain reaction (qRT-PCR) was performed as previously described (Tong *et al.*, 2009). The relative expression level of a target gene was normalized to that of rice *ACTIN1*. All primers used in qRT-PCR are listed in Supplementary Table S1.

### Confocal microscopy

To identify the subcellular localization of REL1, *Agrobacterium* strains separately harbouring pCAMBIA1300-35S-REL1-GFP and pt-rb CD3-1000 plasmids were used to transiently co-express REL1 and pt-rb CD3-1000 in the rice protoplast as previously described (Zhang *et al.*, 2011). The subcellular localization of REL1 and pt-rb CD3-1000 was investigated by confocal microscopy systems. Briefly, GFP fluorescence images were taken using 488-nm laser excitation and the emission was collected via a 525-nm filter. The mCherry images of the pt-rb CD3-1000 were taken using a 561-nm laser.

## Results

### Characterization of the rice *rel1* mutants

**Fig. 1.**
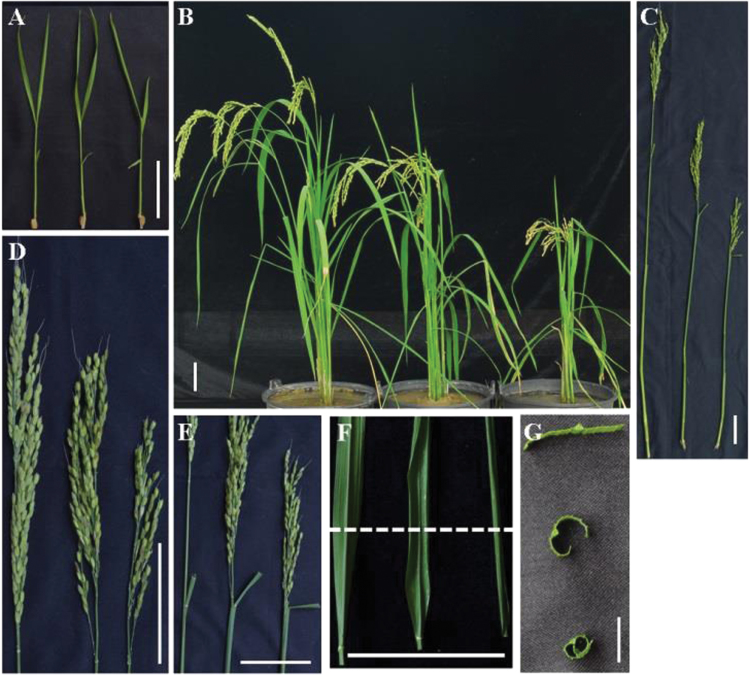
Phenotype of *rel1* mutant. (A) Seedling phenotype of wild type (left), *rel1* heterozygote (middle), and homozygote (right). Seeds were germinated at 28°C for 3 days and then transferred to a paddy field; 10-day-old plants are shown. Bar = 10cm. (B) Mature plant phenotype of wild type (left), *rel1* heterozygote (middle), and homozygote (right). (C–H) The developmental defects in *rel1* were observed in plant height (C), panicle length (D), leaf bending (E), and leaf rolling (F and G). Bar = 10cm. B–F, wild type, *rel1* heterozygote, and homozygote from left to right, respectively. G, wild type, *rel1* heterozygote, and homozygote from top to bottom, respectively.

To identify genes regulating leaf morphology or architecture in rice, a genetic screen was performed with T-DNA insertion lines in rice cultivar ‘Zhonghua 11’ (wild-type plant). A mutant with the most obvious defects in leaf morphology from seedling to the mature stage was then isolated. This mutant was named *rolled and erect leaf 1* (*rel1*). The *rel1* was a dominant mutant (see details below), which did not show any detectable difference compared to wild type during the early growth stage (Fig. 1A). At later growth stages, the *rel1* mutants differed from wild type, showing dwarfism and shortened panicles (Fig. 1B–D). Statistical analyses confirmed that the plant height, panicle length, and numbers of tiller were significantly reduced in *rel1* mutants (Supplementary Fig. S1A–C). The most striking phenotype was the leaf adaxial rolling and bending (Fig. 1E–G). To extensively evaluate the leaf rolling, natural and maximum leaf width were measured and the rolling index (natural width versus maximum width) was used as an indicator for leaf rolling. The maximum width of leaves in wild type and the *rel1* mutant was not different (Supplementary Fig. S1D). However, the natural width of leaves in *rel1* was significantly reduced (Supplementary Fig. S1E), leading to a reduction in rolling index (Supplementary Fig. S1F). These results indicate that the rolling of the *rel1* mutant resulted from changes in the natural width of the leaf rather than changes in the maximum width.

As another predominant feature of *rel1* mutant, leaf bending was measured according to the previously described method (Tong *et al.*, 2009). Leaf bending of *rel1* mutants was approximately 90 degrees, whereas leaf bending in wild type was only 15 degrees (Supplementary Fig. S1G). There were additional defects in the grain of the *rel1* mutant, such as in the grain shape and weight (Fig. 1H; Supplementary Fig. S1H–J). Taken together, these results suggest that *rel1* mutation caused abnormal development in rice, particularly in the leaf morphology.

### Cell structure of *rel1* leaf is altered

**Fig. 2.**
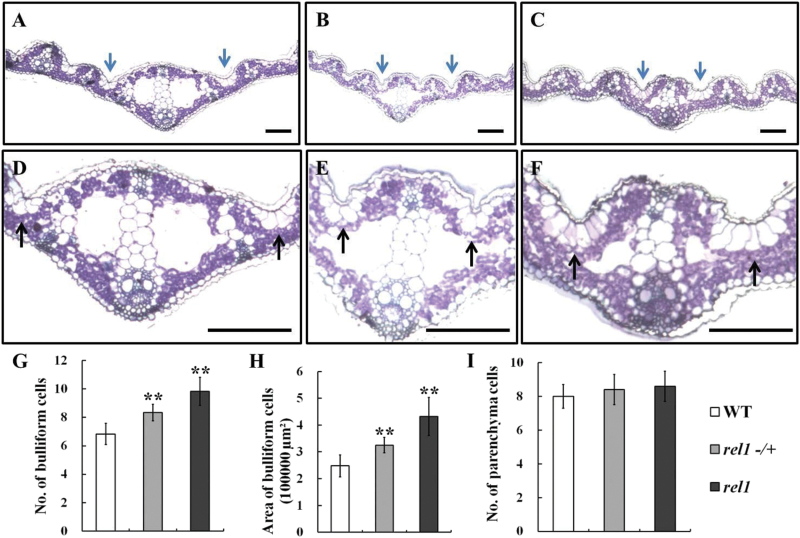
Cell structure is altered in *rel1* mutant. (A–C) Electron microscopy of leaf in wild type (A), *rel1* heterozygote (B), and *rel1* homozygote (C). One-month-old leaves were used in this observation. (D–F) Enlarged view of cell structure of wild type (D), *rel1* heterozygote (E), and *rel1* homozygote (F). Bulliform cells are indicated by arrows and bar = 1cm (A to F). (G–I) Statistical analysis of bulliform cell number (G), bulliform cell size (H), and parenchyma cell number (I). More than 10 samples were investigated, and the significances are indicated by the asterisks at *P* < 0.05 (G and I).

Generally, the adaxial rolling phenotype of mature leaves may be caused by the altered profiles of special kinds of bulky cells on the adaxial epidermis in the leaf, such as the parenchyma and bulliform cells (Botwright *et al.*, 2005; Fujino *et al.*, 2008). Therefore, it was assumed that the rolled leaf phenotype of *rel1* might also be related to changes in the special bulky cells. To test this possibility, cross-sections of mature leaves were observed under an electron microscope. There was no difference between parenchyma cells in wild type and the *rel1* mutant (Fig. 2A–C), whereas the there were discernible differences in the profiles of bulliform cells among the wild type, *rel1* heterozygote (*rel1*−/+), and *rel1* plants (Fig. 2A–C). Enlarged views of the bulliform cells demonstrated significant differences in numbers and size of bulliform cells between wild type and *rel1* (Fig. 2D–F). Statistical results indicated that both numbers and size of bulliform cells in *rel1* mutant were increased (Fig. 2G, H). Therefore, it was concluded that the leaf adaxial rolling phenotype of *rel1* mutants was caused by the altered profiles of bulliform cells.

### Molecular cloning of *REL1*

**Fig. 3.**
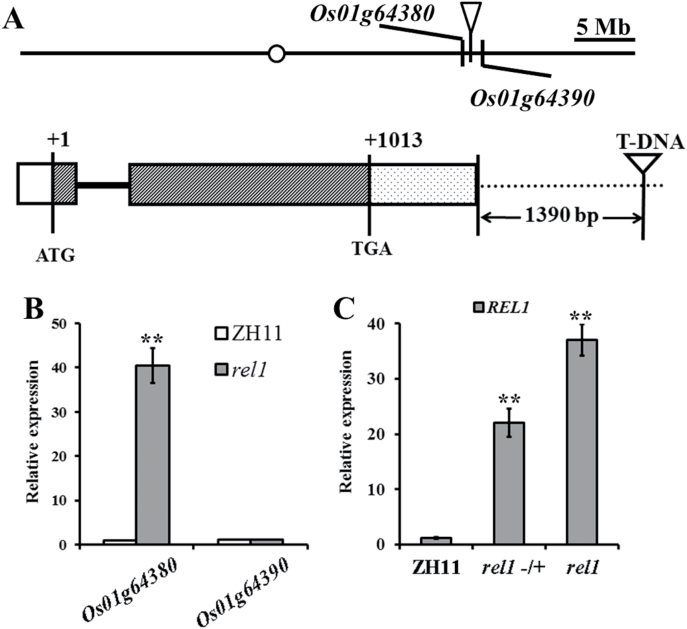
Molecular cloning of the *REL1* gene. (A) Location of T-DNA insertion and the schematic structure of *REL1*. The putative translation start is referred to as +1. ATG, start codon. TGA, stop codon. 5′ UTR, exon, intron, and 3′ UTR are indicated by the white box, black box, solid line, and dotted box, respectively. (B) Relative expression levels of *Os01g64380* and *OS01g64390* were determined by qRT-PCR. (C) Relative expression of *Os01g64380* was determined by qRT-PCR in wild type, *rel1* heterozygote, and *rel1* homozygote. Total RNA was extracted from 1-month-old leaves of corresponding plants (B and C). This experiment was repeated more than three times with similar results, and the values are means ± SD of three biological repeats.

Genetic analysis by backcrossing the *rel1* mutant with wild type indicated that the *REL1* gene was controlled by a single dominant gene, because the segregation of the *rel1*-like, medium, and wild-type phenotype was approximate 1:2:1 (χ^2^ = 0.789). The genomic DNA fragment flanking the T-DNA insertion site in the *rel1* mutant was isolated using thermal asymmetric interlaced PCR. A BLAST search with the flanking sequence indicated that the T-DNA insertion site was 1390bp from the stop codon of *Os01g64380* and 7743bp from the start codon of *Os01g64390* (Fig. 3A), suggesting that the *rel1* phenotype might be caused by an alteration of one of these genes. Co-segregation of *Os01g64380* or *Os01g64390* with *rel1*-like phenotype was then examined in more than 100 F2 individuals. Using special primers (Supplementary Table S1 and Supplementary Fig. S2), the *rel1* phenotype was found to fully co-segregate with *Os01g64380*. In addition, qRT-PCR analysis demonstrated that the transcript level of *Os01g64380* was dramatically elevated in *rel1*, while that of *Os01g64390* remained unchanged, as compared to wild type (Fig. 3B). Moreover, the higher expression level of *Os01g64380* was also detected in *rel1* heterozygous plants compared to wild type, and the highest expression was shown in the *rel1* homozygote (Fig. 3C). These results suggest that the phenotype of *rel1* might be caused by the elevated expression of *Os01g64380*.

According to the annotation in the Rice Genome Annotation Project website (http://rice.plantbiology.msu.edu/), *REL1* comprises two exons and one intron and encodes an unknown protein (Fig. 3A). Interestingly, REL1 protein is highly conserved in monocot plants (Supplementary Fig. S3). Therefore, it is suggested that REL1 is a functional unknown protein that is only present in monocot plants.

### Knockdown of *REL1* in the *rel1* mutant restores the wild-type phenotype

**Fig. 4.**
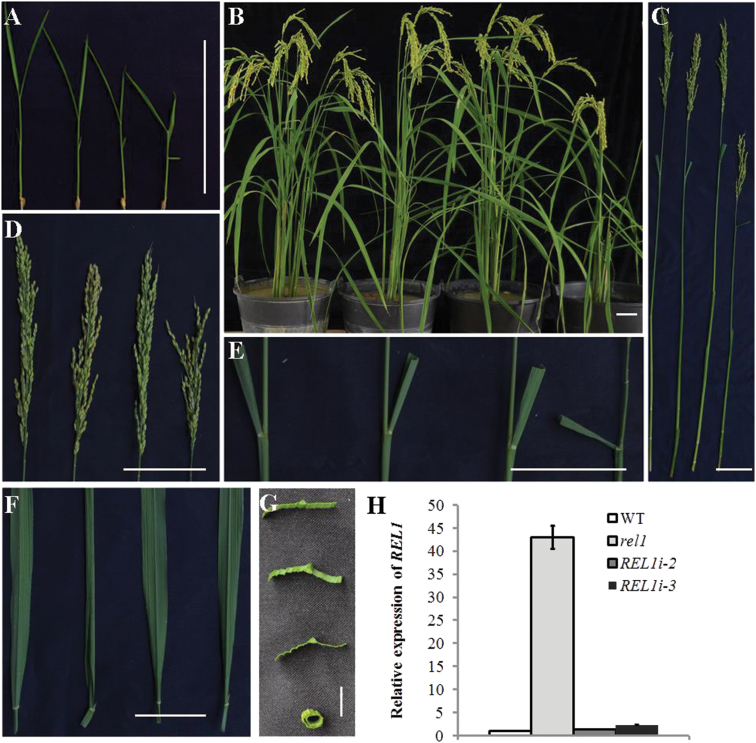
Knockdown of *REL1* in *rel1* restored the defects of *rel1* mutant. (A) Seedling phenotype of wild type (left), *REL1i* plants (*REL1i-2* and *REL1i-3*, the middle two plants), and *rel1* mutant (right). Two-week-old plants are shown. Bar = 10cm. (B) Mature plant phenotype of wild type (left), *REL1i* plants (*REL1i-2* and *REL1i-3*, the middle two plants), and *rel1* mutant (right). (C–) The defect phenotype of *rel1* was restored by knockdown of *REL1* for plant height (C), panicle height (D), leaf bending (E), and leaf rolling (F and G). Bar = 10cm. C–F, wild type, *REL1i-2*, *REL1i*-3, and *rel1* mutant from left to right, respectively. G, wild type, *REL1i-2*, *RELIi-3* and *rel1*from top to bottom, respectively. (H) Relative expression levels of *REL1* in wild type, *rel1* mutant, and *REL1* antisense plants. One-month-old leaves were used for the qRT-PCR analysis, and the values are means ± SD of three biological repeats.

To verify the identity of *REL1*, a *REL1* antisense construct driven by the ubiquitin promoter was produced and introduced into *rel1* mutants via *A. tumefaciens*-mediated transformation. Eight independent lines (*REL1i*) were obtained and all of them displayed a similar phenotype to the wild type. Among the eight transgenic lines, *REL1i-2* and *RELi-3* were selected for the following studies owing to the most significant repression of *REL1* (Fig. 4H). Detailed analyses showed that the plant architecture and leaf morphology of the *rel1* mutant phenotype, including plant height and panicle length, as well as leaf bending and rolling, were restored in both *REL1i-2* and *RELi-3* lines (Fig. 4A–G). Interestingly, knockdown of *REL1* in wild type did not show any detectable mutant phenotype (Supplementary Fig. S4A–D). This might be owing to the low expression level of endogenous *REL1* in wild type (Supplementary Fig. S4E). Taken together, these results indicate that *REL1* was the target gene and played a positive role in leaf rolling and bending in rice.

### Overexpression of *REL1* leads to a *rel1*-like phenotype

**Fig. 5.**
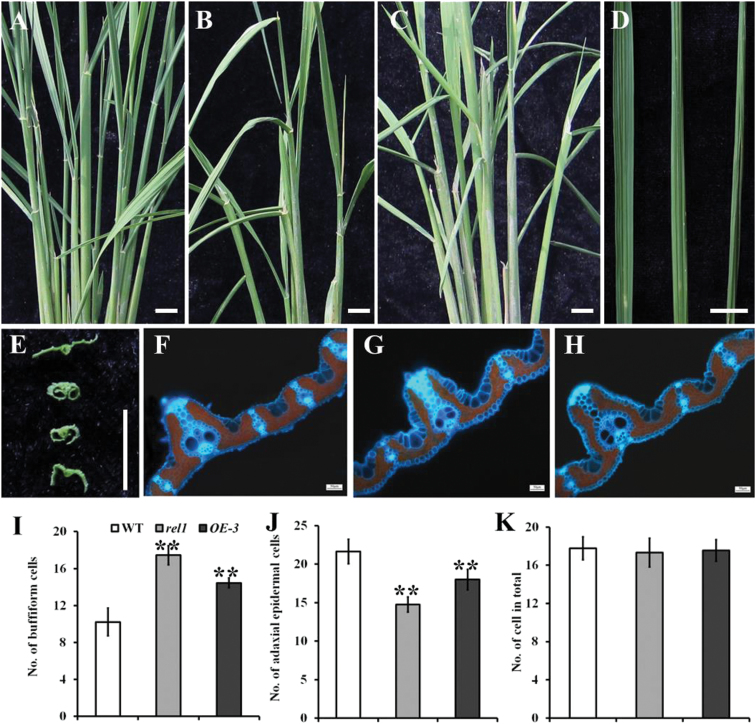
Overexpressing *REL1* results in a *rel1*-like phenotype. (A–C) Leaf morphology of wild type (A), *rel1* mutant (B), and the *REL1* overexpressing plant (*OE-3*, C) at tiller stage. Two-month-old plants are shown. (D) Leaf phenotype of wild type (left), *rel1* (middle), and *OE-3* (right). (E) Rolled leaf phenotype of wild type, *rel1*, *OE-3*, and *OE-4* from top to bottom. (F–H) The cell structure of leaves in wild type (F), *rel1* (G), and *OE-3* (H). One-month-old leaves were used in this experiment. (I–K) Statistical analysis of bulliform cell number (I), bulliform cell size (J), and parenchyma cell number (K) in wild type, *rel1*, and *OE-3*. More than 10 samples were investigated, and the significances are indicated by the asterisks with *P* < 0.05 (I and J). Bar = 10cm (A to E).

To further investigate whether activating *REL1* would induce a *rel1*-like phenotype, a *REL1* overexpression construct driven by the ubiquitin promoter was produced and introduced into wild type via *A. tumefaciens*-mediated transformation. Six independent transgenic lines (*OE*) were obtained, and all of them exhibited upregulated trends of *REL1* expression (Supplementary Fig. S5A). Because the *OE-3* line showed the highest expression among the transgenic lines (Supplementary Fig. S5A), it was selected for the following studies. Consistent with the expression levels, *OE-3* plants displayed almost the same phenotype as the *rel1* mutant, including fewer tillers (Fig. 5A–C) and rolled leaf phenotype (Fig. 5D, E). Erect leaf and dwarfism were also observed in *OE-3* plants (Supplementary Fig. S5B–F). The numbers of bulliform cell were increased in *OE-1* in comparison with the wild type (Fig. 5F–5I). Statistical analysis confirmed the significant reduction of the numbers of adaxial epidermal cells in *rel1* and *OE-3* (Fig. 5J). However, the numbers of parenchyma cells in *rel1* and *OE-3* leaves were similar to those in wild-type leaves (Fig. 5K). Taken together, it is concluded that activated *REL1* induces the *rel1*-like phenotype and triggers cell division and expansion of bulliform cells.

### Expression pattern of *REL1*

**Fig. 6.**
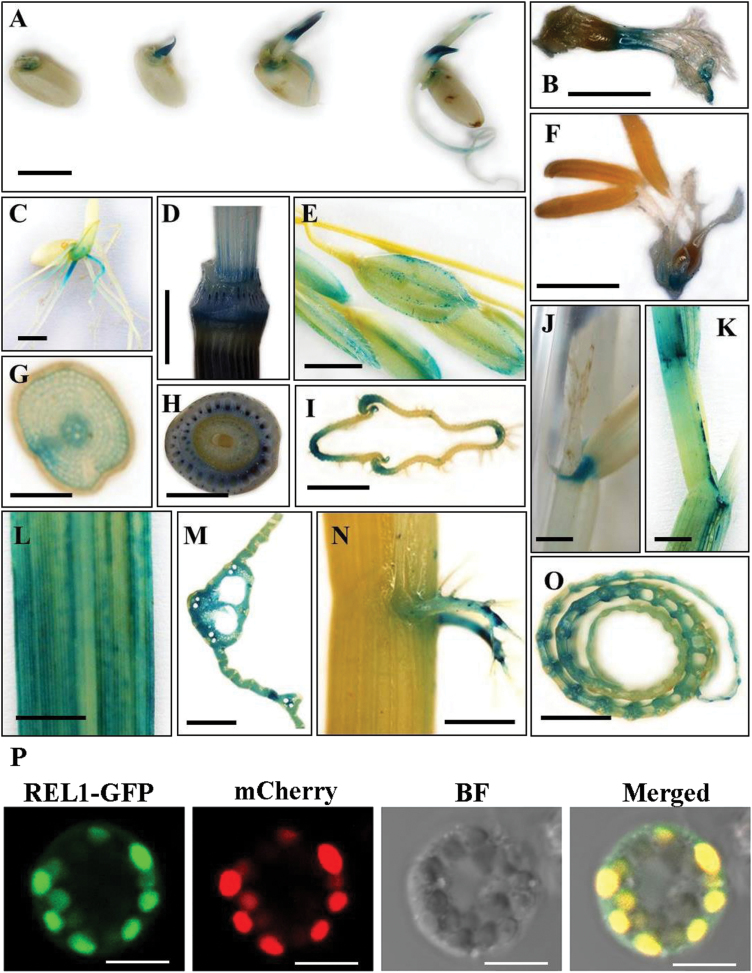
Expression pattern of *REL1*. (A) GUS staining of seedling. The signals were revealed by promoter–GUS fusion analysis in transgenic plants. (B–F) GUS activity was observed in the stamen (B), root (C), stem (D), spikelet (E), and inflorescence (F). (G–I) Cross-section of C, D, and E reveals the specific pattern of *REL1*. (J–O) GUS signals were observed in ligule (J), sheath and blade (K), mature leaf (L), cross view of mature leaf (M), lamina joint (N), and cross view of culm (O). A to O, bar = 1cm. GUS staining was performed in more than three independent lines with similar patterns. (P) Transient co-expression of REL1-GFP fusion protein and a plastid marker pt-rb CD3-1000 (Nelson *et al.*, 2007) in rice protoplasts revealed that REL1 is mainly located in the plastid. The pt-rb CD3-1000 was visualized by mCherry fluorescence. BF, bright field. Bars = 10 µm.

To elucidate the expression pattern of *REL1*, the 2545bp endogenous promoter of *REL1* was fused with GUS and the resulting construct was then introduced into the wild type. GUS staining results demonstrated that *REL1* was specifically expressed in shoot apical meristem during seed germination (Fig. 6A). In the inflorescence, the GUS signals of *REL1* were stained in the stamens but not in the pistil (Fig. 6B, F). Interestingly, *REL1* was also expressed in the elongation zone of roots at the first leaf stage (Fig. 6C), and further detected in the epidermis and endodermis (Fig. 6G). At the heading stage, *REL1* was subsequently observed in the stem (Fig. 6D), and GUS staining was specifically detected in the endodermis (Fig. 6H). At the mature stage, there was an obvious GUS signal of *REL1* on the spikelet hull (Fig. 6E), in which *REL1* was only found in a portion of the lemmas and paleas (Fig. 6I). Further investigations showed that *REL1* was constitutively expressed in the leaf during growth and development (Fig. 6J, K, L), particularly in the ligule, leaf sheath, and vascular (Fig. 6K, M, O). *REL1* was also expressed in the lamina joint (Fig. 6N). The GENEVESTIGATOR tool was also used to analyse the expression pattern of *REL1*. As shown in Supplementary Fig. S6, *REL1* was constitutively expressed in the root, leaf, and inflorescence, and especially accumulated in the mature leaf, which is consistent with the GUS staining results.

To explore the subcellular localization of REL1 protein, REL1 was fused in-frame to the *GFP* gene. The resulting *REL1*-*GFP* transgenes, under the control of the Cauliflower mosaic virus 35S (35S) promoter, was transiently co-expressed with a plastid marker pt-rb CD3-1000 (Nelson *et al.*, 2007) in rice protoplasts. Results indicated that REL1-GFP localized in the plastid (Fig. 6P). Taken together, it appears that *REL1* is constitutively expressed in various tissues and stages and encodes a plastid-localized protein.

### Expression of BR-associated genes is altered in *rel1* mutant

**Fig. 7.**
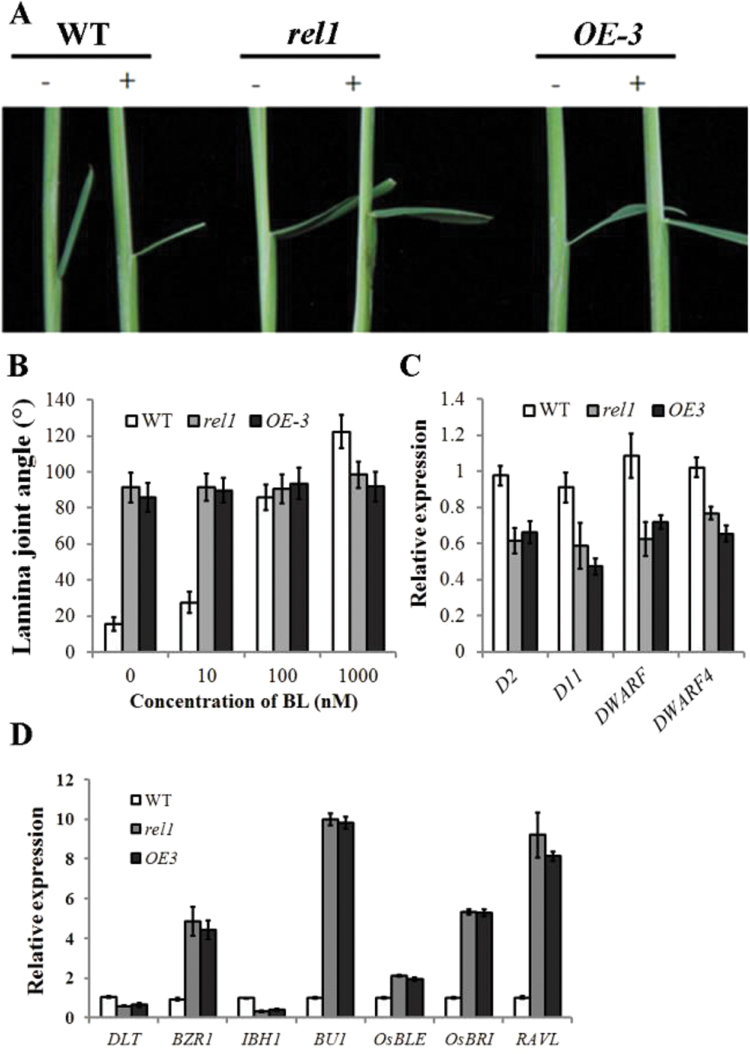
BR response of *REL1*. (A) Phenotype of wild type, *rel1*, and *OE-3* with or without BR treatment. Two-week-old plants were used in the micro-drop treatments. The BR treatment was performed as previously described (Tong *et al.*, 2009). Bar = 10cm. (B) Quantification of lamina joint bending angle in wild type, *rel1*, and *OE-3* with various concentration of BL treatment. Bars indicate standard deviation (n = 20). (C) Relative expression of BR biosynthetic genes *D2*, *D11*, *DWARF4*, and *DWARF* in wild type, *rel1*, and *OE3*. (D) Relative expression of BR signalling genes in wild type, *rel1*, and *OE3*, respectively. C and D, the values are the mean ± SD with three biological replicates.

BR plays a vital role in plant growth and development, especially in leaf morphology. BR-deficient mutants usually exhibit a rolled and/or erect leaf phenotype (Bishop and Yokota, 2001; Hong *et al.*, 2004). Considering the rolled and erect leaf phenotype present in the *rel1* mutant, the question was asked whether the BR pathway is eliminated in *rel1*. To address this issue, the BR response of the *rel1* mutant was examined by a lamina inclination assay as described previously (Tong *et al.*, 2009). As expected, the wild-type plant was hypersensitive to BR hormone and showed the erect leaf phenotype after BR treatment (Fig. 7A). Notably, BL treatment induced a lamina joint angle in wild type of above 120 degrees at a concentration of 1000nM (Fig. 7B). By contrast, there was no change in the lamina joint angle of either *rel1* or *OE-3* with any concentration of BL treatment (Fig. 7A, B), indicating that *rel1* and *OE-3* were insensitive to the BR. qRT-PCR analysis of BR-associated genes was performed to evaluate the BR response to *rel1*. The *D2*, *D11*, and *DWARFs* have been well characterized as BR biosynthetic genes in both *Arabidopsis* and rice (Choe *et al.*, 2001; Hong *et al.*, 2003; Tanabe *et al.*, 2005; Kim *et al.*, 2006). Results showed that all of them were slightly downregulated in the *rel1* and *OE-3* lines (Fig. 7C). These results suggest that *REL1* was not tightly associated with BR biosynthesis. Accumulating evidence implicates that inhibition of *OsBZR1* leads to erect leaf phenotype, whereas *OsBZR1*-mediated leaf morphology is controlled by *DLT* (Tong *et al.*, 2009). Surprisingly, the qRT-PCR analysis demonstrated that *OsBZR1* was significantly upregulated in *rel1* and *OE-3*, with ~5-fold changes (Fig. 7D), whereas *DLT* was repressed in *rel1* and *OE-3*, along with another downstream regulator *IBH1* (Fig. 7D). In addition, other BR signalling genes, including *BU1*, *OsBRI*, and *RAVL*, were significantly upregulated (Fig. 7D). Taken together, it is proposed that *REL1* coordinates the expression of BR signalling-related genes to regulate leaf morphology.

### Identification of REL1-interacting proteins by a yeast two-hybrid screen assay

To further explore the biological function of REL1, a yeast two-hybrid screen assay was performed to identify REL1-interacting proteins (RIPs). In total, 51 positive clones were obtained. However, only seven were confirmed to encode annotated proteins. Among these *RIPs*, both *RIP1* and *RIP2* encode zinc finger proteins, and the RIP3 protein belongs to an expansin-like A subfamily, while *RIP4*, *RIP5*, *RIP6*, and *RIP7* encode hydrolase, oxidase, thaumatin and an unknown protein, respectively (Table 1). To further verify the relationship between REL1 and RIPs, knockdown of *RIPs* in the *rel1* mutant background was performed. RIP3 protein was selected as a representative case, because a study in *Nicotiana tabacum* has implicated that upregulation of an expansin protein could induce the formation of leaf curvature (Sloan *et al.*, 2009). Phenotypic analyses on the *rip3 rel1* double mutants illustrated that knockdown of *RIP3* in *rel1* could rescue the *rel1* phenotype, including plant height, leaf rolling, and leaf bending (Supplementary Fig. S7). Therefore, it is proposed that *RIPs*, particularly *RIP3*, are involved in regulating rice development through interplay with *REL1*.

**Table 1.** List of REL1-interacting proteins from yeast 2-hybrid screen

Name	Gene locus	Gene annotation^a^
*RIP1*	LOC_Os06g17410	dof zinc finger protein
*RIP2*	LOC_Os10g30850	zinc finger protein
*RIP3*	LOC_Os03g04020	Expansin-like A subfamily
*RIP4*	LOC_Os01g71340	glycosyl hydrolase
*RIP5*	LOC_Os06g37150	putative L-ascorbate oxidase
*RIP6*	LOC_Os12g43380	thaumatin-like protein
*RIP7*	LOC_Os03g16950	uncharacterized protein

^a^ Gene annotation was obtained from the GRAMENE or NCBI database.

## Discussion

Leaves plays a vital role in photosynthesis, respiration, and transpiration for plant growth and development (Govaerts *et al.*, 1996). Establishment of moderate leaf rolling is considered an important agronomic strategy in rice that can increase photosynthesis and reduce transpiration (Lang *et al.*, 2004; Zhang *et al.*, 2009; Zou *et al.*, 2011), thereby increasing rice yield. In the present study, a dominant mutant, named *rel1*, was characterized. This mutant displayed a rolled and erect leaf, dwarfism, and small grain phenotype, eventually resulting in a reduction in grain yield. Overexpression of *REL1* resulted in a *rel1*-like phenotype, indicating that *REL1* positively participates in regulating leaf morphology. The results also suggested that *REL1* regulates leaf bending through coordination of the BR pathway.

Generally, leaf rolling is induced by water loss from bulliform cells on the leaf upper epidermis in rice (O’Toole J and Cruz, 1980), suggesting that the number and density of bulliform cells may affect the extent of leaf rolling. However, it has been demonstrated that water loss from the adaxial subepidermal sclerenchyma and mesophyll also contributes to leaf rolling, and leaf rolling can also occur in leaves lacking bulliform cells (Shields, 1951). In the present study, *rel1* mutants displayed abaxially rolled leaves, which resulted from the increased number and size of bulliform cells on the abaxial side of leaf blades (Fig. 2). Bulliform cells in *rel1* were larger than those in the wild type, which may alter the mechanical properties of the leaf abaxial surface. Knockdown of *REL1* expression in a *rel1* background restored a wild-type phenotype (Fig. 4). Consistent with the activation of *REL1* in *rel1* mutants, overexpression of *REL1* in wild type also resulted in a *rel1*-like phenotype (Fig. 5), indicating the positive role of *REL1* in controlling leaf rolling via the regulation of cell division and expansion of bulliform cells. However, this positive role of *REL1* in regulating leaf morphology needs to be further understood.

A bioinformatics analysis suggested that *REL1* is specific to monocot plants because the REL1 protein homologies were only found in monocots such as rice, *Zea mays*, and *Sorghum bicolor* (Supplementary Fig. S3). In addition, BLAST analysis indicated that there was no relevant domain or motif present in REL, suggesting that *REL1* encodes a functional unknown protein that only exists in monocot species. Considering the leaf architecture and morphology of monocot plants, it can be speculated that the function of these REL-like proteins may share some degree of conservation in leaf development in monocotyledonous plants, although some differentiations might also have occurred.

BR has been shown to participate in plant growth and development, especially in determining leaf morphology. BR-deficient mutants always exhibit a dwarf, erect, and rolled leaf phenotype (Bishop and Yokota, 2001; Hong *et al.*, 2004). Results here indicated that BR biosynthesis-related genes were slightly reduced in the *rel1* and *OE-3* lines (Fig. 7), suggesting that *REL1* likely is not associated with BR biosynthesis. However, *OsBZR1* and *DLT*, which control bending of the lamina joint in rice (Tanaka *et al.*, 2009; Tong *et al.*, 2009; Tong *et al.*, 2012), were induced and repressed, respectively, in *rel1* and *OE-1* (Fig. 7). These findings differed from the previous discovery that erect leaf phenotype results from the inhibition of *BZR1* (Hong *et al.*, 2004; Sakamoto *et al.*, 2006; Bai *et al.*, 2007). Additionally, other BR signalling genes, including *BU1*, *OsBRI*, and *RAVL*, were upregulated in the *rel1* and *OE3* lines (Fig. 7).Therefore, it is tempting to further investigate how *REL1* coordinates the BR signalling genes to regulate leaf morphology in rice.

Because there is little knowledge about the biological function of REL1, a yeast two-hybrid was used to screen the RIPs. Only seven candidates were identified and confirmed. Among the corresponding seven *RIPs*, three were of more interest, *RIP1* and *RIP2* encoding two zinc finger proteins, and *RIP3* encoding an expansin-like protein. Some regulators of leaf morphology have previously been identified as zinc finger protein. For example, the *LIC* gene encoding a zinc finger protein is involved in the regulation of leaf morphology (Zhang *et al.*, 2012). Preliminary results here indicated that knockdown of *RIP1* or *RIP2* in *rel1* would rescue the defect leaf phenotype of *rel1* (data not shown). Therefore, it is proposed that both RIP1 and RIP2 may be involved in regulating leaf morphology through their interaction with REL1. Expansin proteins were initially identified as cell wall proteins capable of promoting the extension of plant tissue *in vitro* (McQueen-Mason *et al.*, 1992). Studies in tobacco demonstrated that induction of expansin proteins could result in leaf curvature (Sloan *et al.*, 2009). In the present study, the relationship between REL1 and RIP3 was further verified by knockdown of *RIP3* in the *rel1* mutant background, and a phenotypic analysis demonstrated that this knockdown elevated the expression of *RIP3* in *rel1* and could restore the wild-type phenotype (Supplementary Fig. S7). These results suggest that REL1 interacts with RIPs, particularly RIP3, to regulate leaf development. However, how REL1 interplays with these RIPs in regulating leaf development and other traits still remains to be further elucidated.

In summary, a novel important regulator, *REL1*, involving in regulating leaf morphology has here been characterized. Results indicate that *REL1* positively controls leaf rolling and bending, and may coordinate BR-associated genes to regulate leaf morphology. In addition, a yeast 2-hybrid screen revealed seven REL-interacting proteins, which may improve our understanding of the role of REL1 in regulating other traits in rice, such as the control of tiller, plant height, and grain shape.

## Supplementary data

Supplementary material is available at *JXB* online.

Supplemental Fig. S1. Statistical analysis of agronomic traits in *rel1* mutants.

Supplemental Fig. S2. Genotyping of wild type, *rel1* heterozygote, and *rel1* homozygote mutants.

Supplemental Fig. S3. Alignment of REL1-like proteins among monocot plants.

Supplemental Fig. S4. Knockdown of *REL1* in wild type did not result in a mutant phenotype.

Supplemental Fig. S5. Overexpression of *REL1* leads to a *rel1*-like phenotype.

Supplemental Fig. S6. Expression pattern of *REL1* in various tissues and stages.

Supplemental Fig. S7. Knockdown of *RIP3* in *rel1* is capable of rescuing *rel1* mutant phenotype.

Supplemental Table S1. Primers used in this study.

Supplementary Data
