# Supplementary material for: Characterization of Rolled and Erect Leaf 1 in regulating leave morphology in rice
Source: J Exp Bot. 2015 Jul 2;66(19):6047–58. doi: 10.1093/jxb/erv319 (PMC4566990; doi:10.1093/jxb/erv319)
Supplement: Supplementary Data [file supp_66_19_6047__index.html]

Characterization of Rolled and Erect Leaf 1 in regulating leave morphology in rice — Supplementary Data 

# Characterization of *Rolled and Erect Leaf 1* in regulating leave morphology in rice

## Supplementary Data

Data files

- Supplementary Data - Supplementary Data
