# Supplementary material for: Characterization of Rolled and Erect Leaf 1 in regulating leave morphology in rice
Source: J Exp Bot. 2015 Jul 2;66(19):6047–58. doi: 10.1093/jxb/erv319 (PMC4566990; doi:10.1093/jxb/erv319)
Supplement: Supplementary Data [file supp_erv319_REL1_Suppl_materials.pdf]

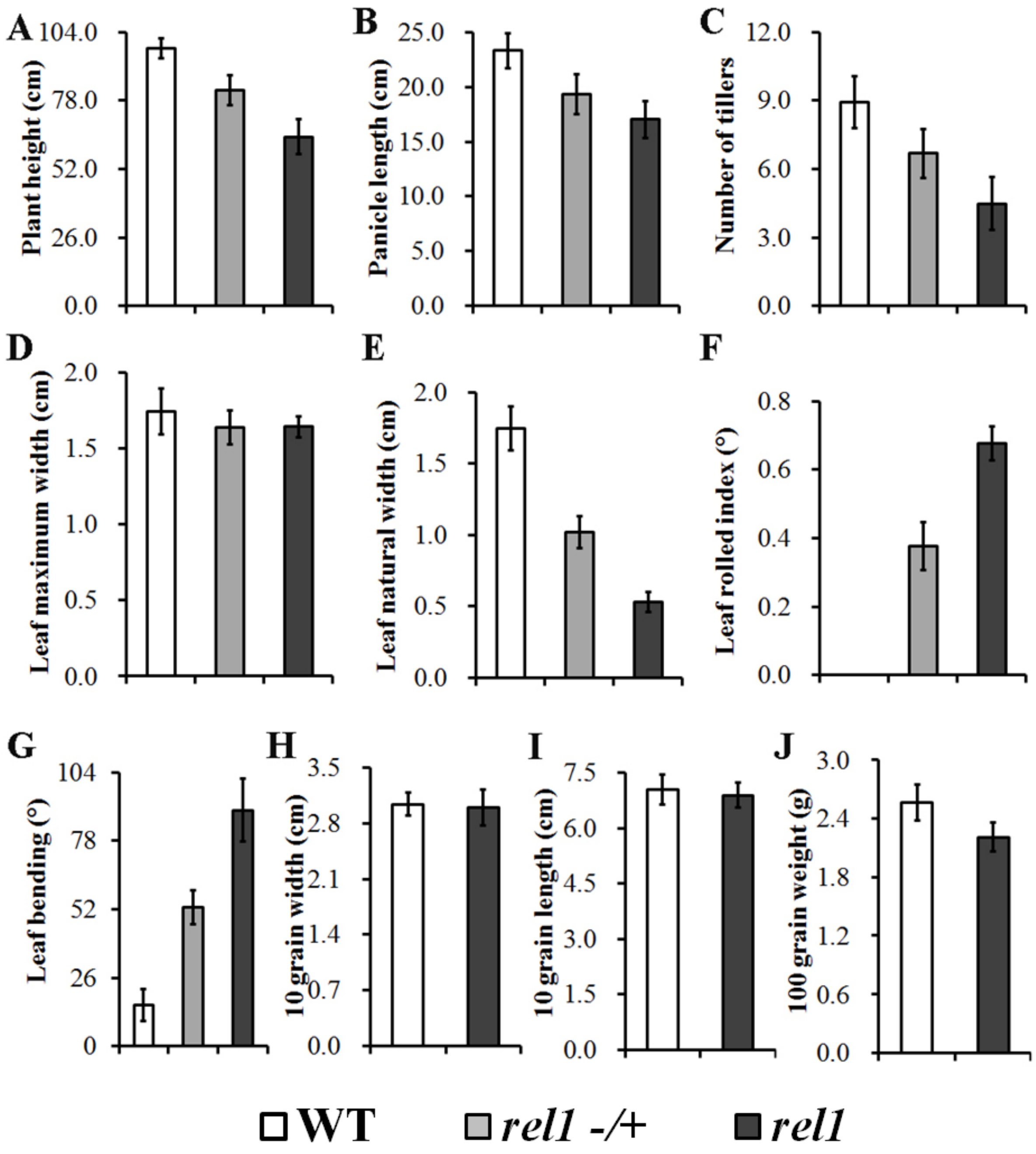

Supplemental Figure S1. Statistical analysis of agronomic traits in *rel1* mutants. A to G, Statistical analysis of wild type, *rel1* heterozygote (*rel1* -/+) and homozygote (*rel1*) mutants in plant height (A), panicle length (B), number of tillers (C), leaf maximum width (D), leaf natural width (E), leaf rolling index (F) and leaf bending (G). More than 20 plants were used in statistic at flowering period. H to J, Statistical analysis of grain width (H), grain length (I) and grain weight (J) in wild type and *rel1* mutants. Seeds from more than 10 plants were used after harvest.

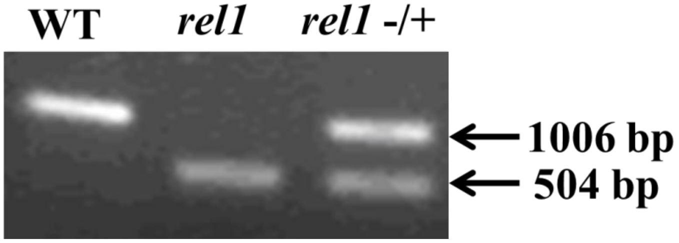

Supplemental Figure S2.Genotyping of wild type, *rel1* heterozygote and homozygote mutants. The one-weeks-old leaves from wild type, *rel1* heterozygote and homozygote were used for genotype identification.

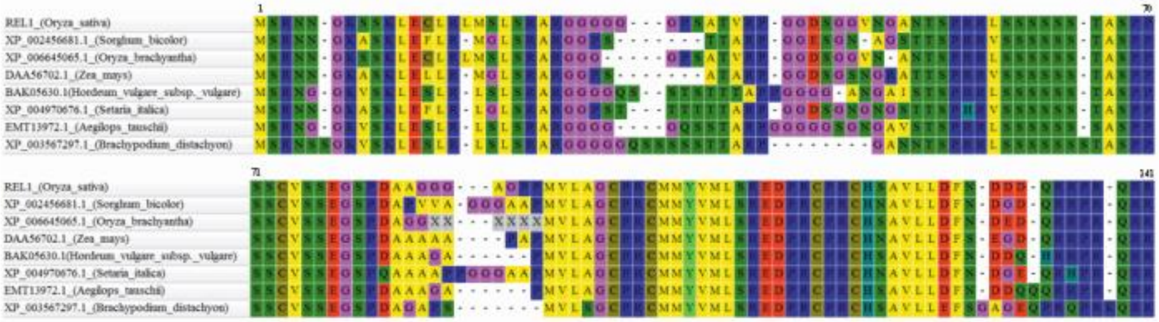

Supplemental Figure S3.Alignment of REL1-like proteins among monocot plants. Sequence alignment of the REL1-like proteins with various monocot plants by using MEGA 6.06 software. The NCBI accession number and species of REL1-like proteins are listed on the parentheses.

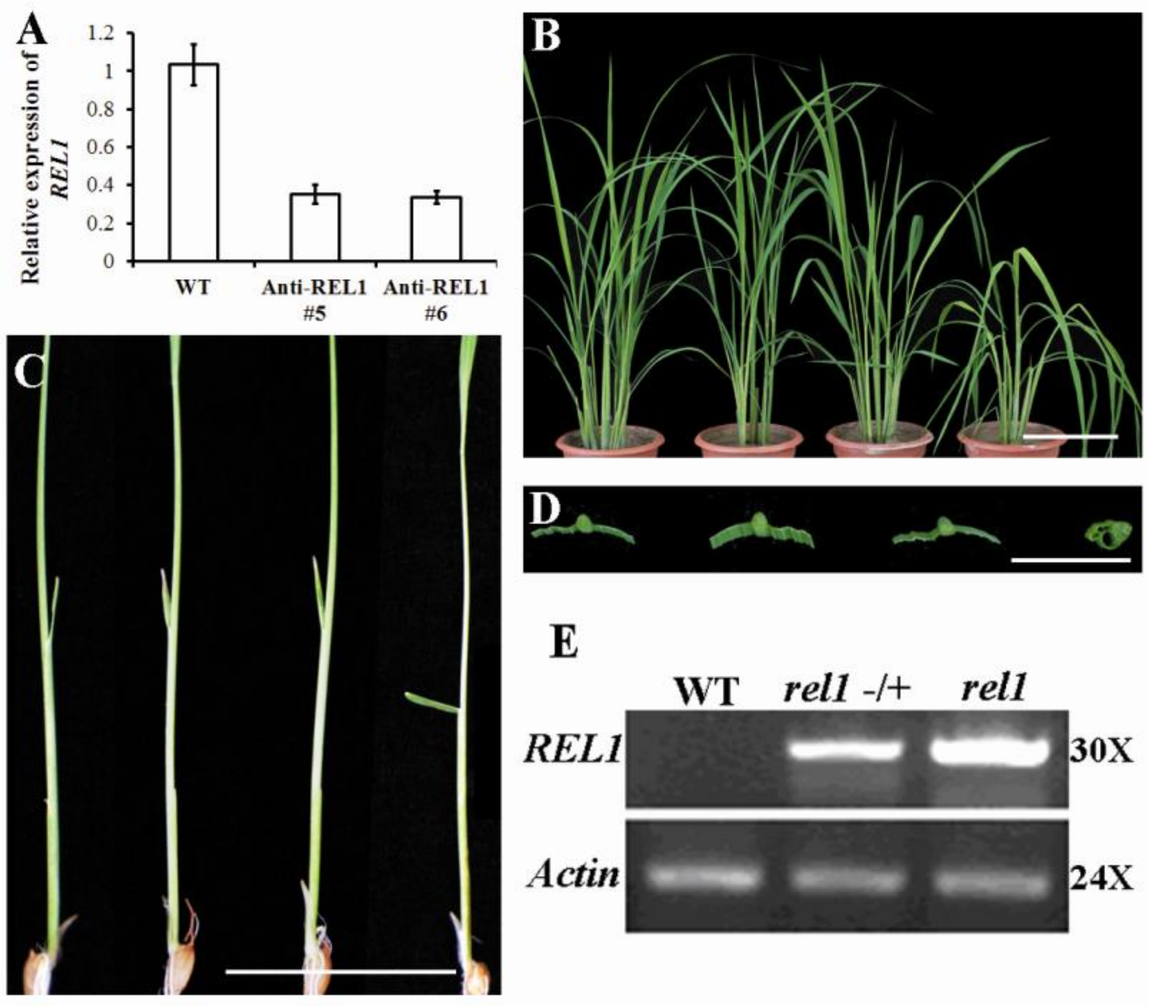

Supplemental Figure S4.Knockdown of *REL1* in wild type incapable of showing apparent phenotype. A, Relative expression of *REL1* in WT, anti-REL1 line 5 and 6, and *rel1* mutant. B, Phenotype of wild type (left), Anti-*REL1* (transgenic line 5 and 6, middle) and *rel1* mutant (right). Two-months-old plants were observed. C, Leaf bending phenotype of wild type (left), Anti-*REL1* (transgenic line 5 and 6, middle) and *rel1* mutant (right) seedlings. D, Leaf section of the (B) with the wild type (left), Anti-*REL1* (transgenic line 5 and 6, middle) and *rel1* mutant (right). E, expression of *REL1* in wild type, *rel1* heterozygote (*rel1* -/+) and homozygote was determined by the semi quantitative RT-PCR. Total RNA was extracted from two-weeks-old leaves, and this experiment repeated more than three biological repeats. X, PCR cycles. B and C, bar is 10 cm. D, bar is 1 cm.

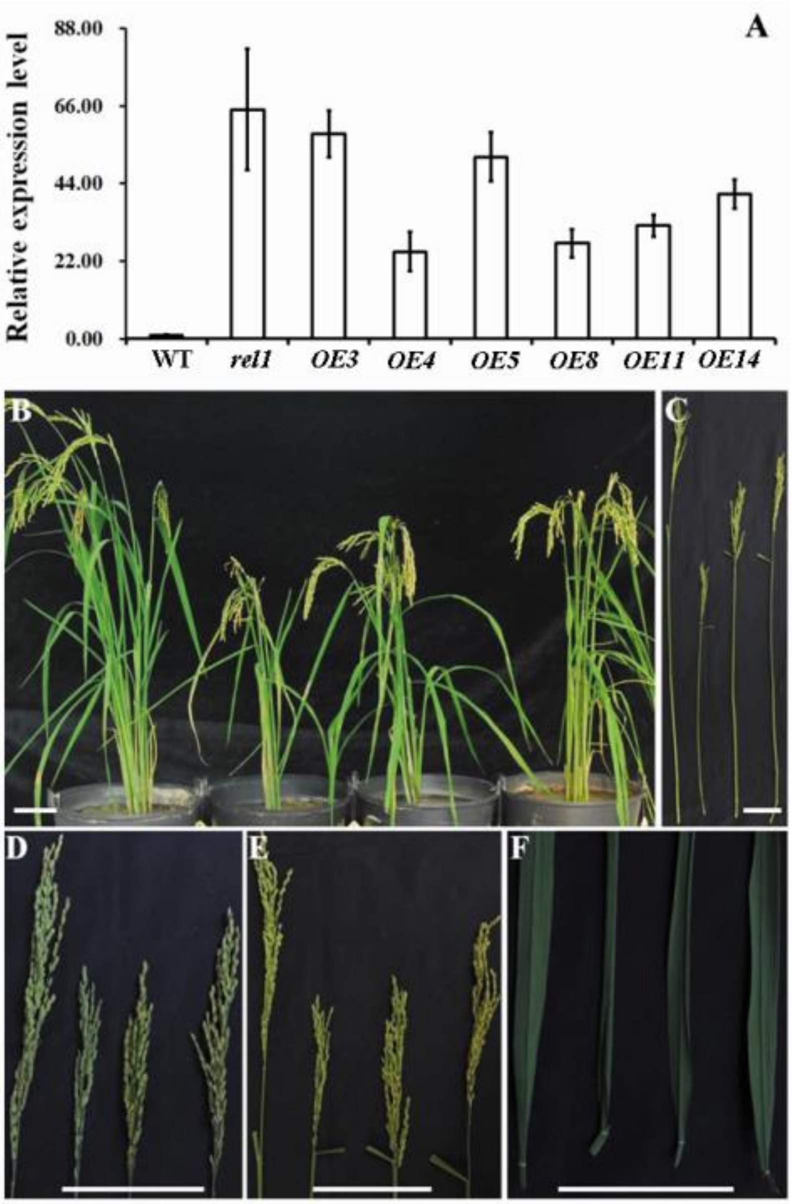

Supplemental Figure S5.Overexpression of *REL1* leads to a *rel1*-like phenotype. A, Identification of *REL1* overexpressing plants. The relative expression levels of *REL1* in wild type, *rel1* mutant and *OEs* were determined by qRT-PCR. Two-weeks-old leaves were used in this experiment. Values are the means ± SD with more than three biological repeats. B, Phenotype of wild type (left), *rel1* mutant (middle) and *OEs* (*OE-3* and *OE-5*, right) at the heading stage. C to F, Observation of agronomic traits of *OE* plants in plant height (C), panicle length (D), leaf bending (E) and leaf rolling (F). From left to right is wild type, *rel1*, *OE-3* and *OE-5*, respectively. B to F, Bar = 10 cm.

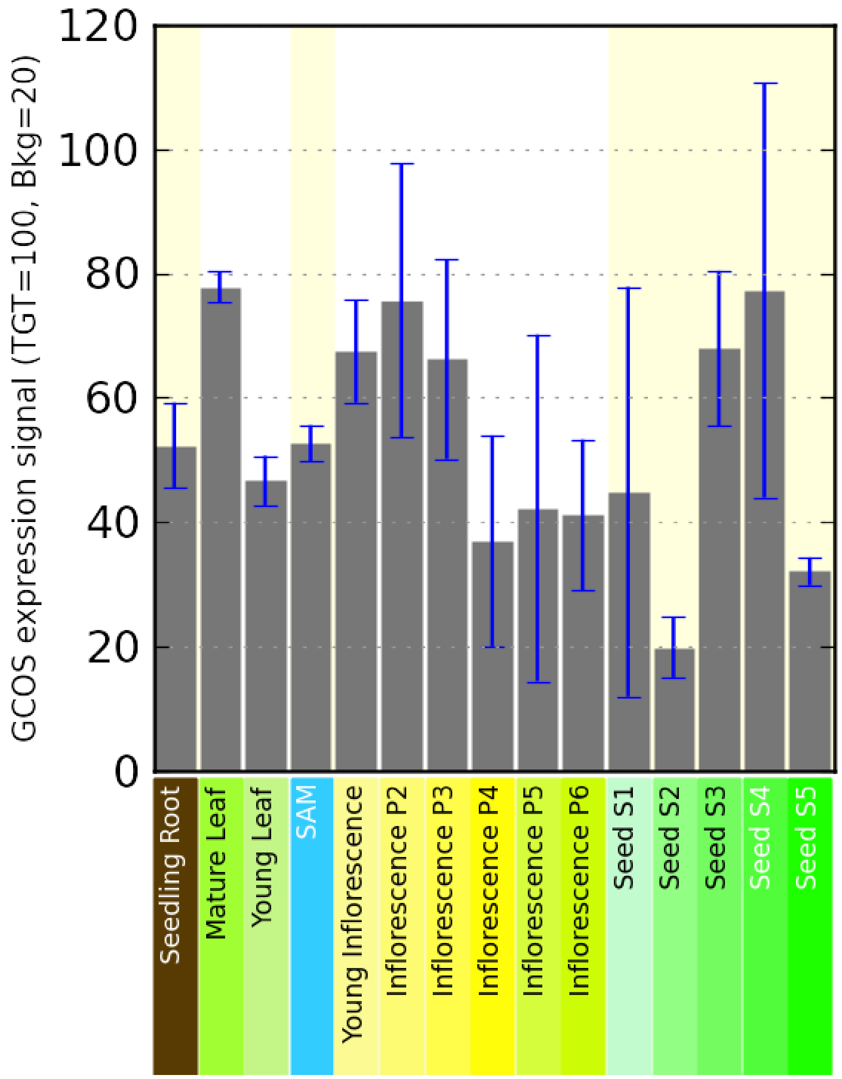

Supplemental Figure S6. Expression pattern of *REL1* in various tissues and stages. The expression values of *REL1* was taken from the microarray collection at the BAR website (http://bar.utoronto.ca/interactions/). The chart shows the relative expression of *REL1* in the different tissues and stages (colorful panels at the bottom).

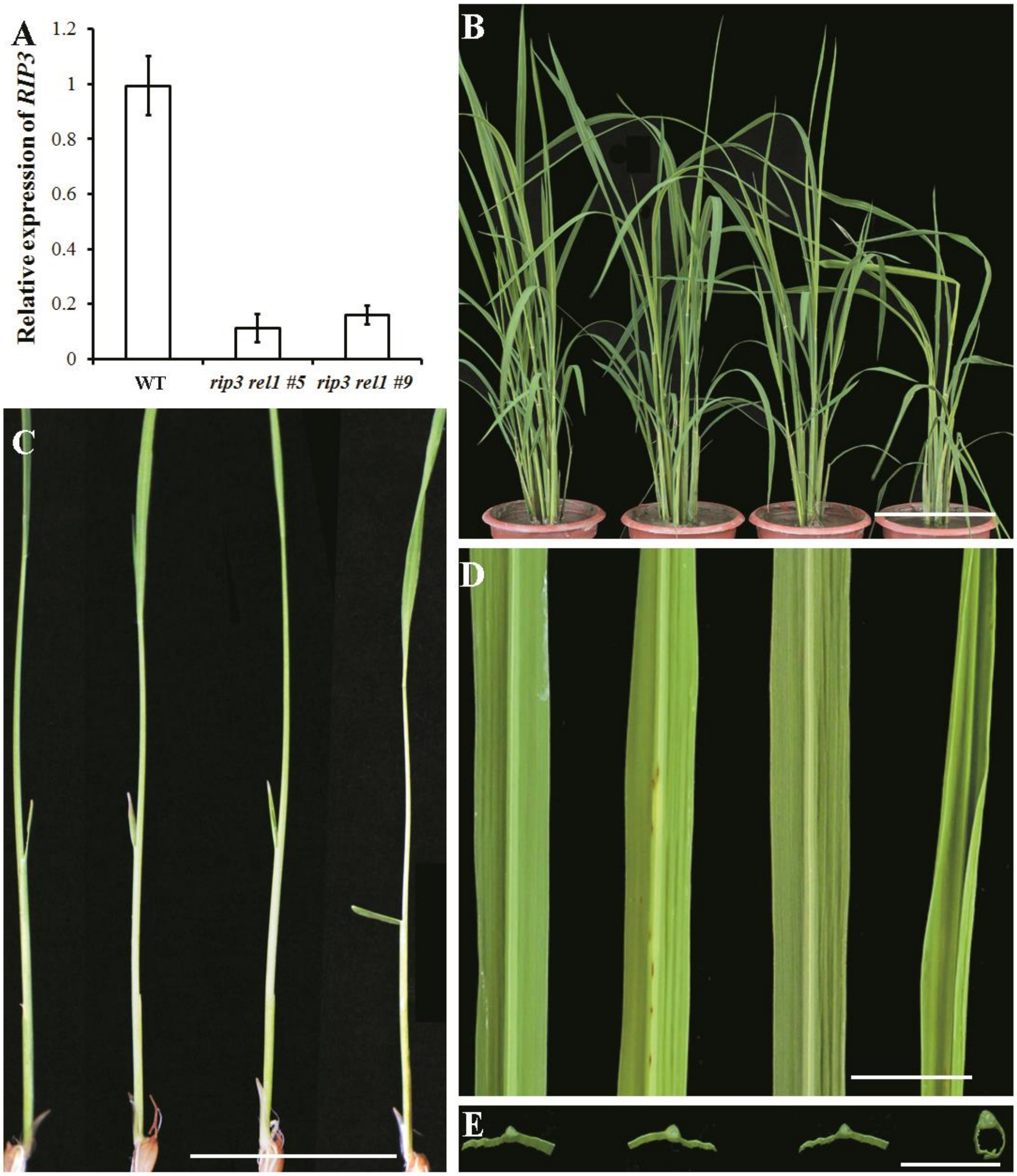

Supplemental Figure S7. Knockdown of *RIP3* in *rel1* capable of rescuing *rel1* mutant phenotype. A, Relative expression of *RIP3* in the wild type and *rip3 rel1* double mutants. B to E, Phenotype of wild type, *rel1* and *rip3 rel1* double mutant in the tiller stage (B), leaf bending in the seedling stage (C), leaf rolling from tiller stage (D) and leaf section (E) which was derived from (D). B to E, from left to right is wild type, *rip3 rel1* line 5 and line 9, and *rel1* mutant, respectively. B and C, bar is 10 cm. D and E, bar is 1 cm.

Supplementary Table S1. Primers used in this study.

Experiments	Name	Sequence of Primers (5'-3')	Annotation/vector
Flanking of T-DNA of *rel1*	*TR1*	5′-ATgCCATCATTgCgATAAAgg-3′	T-DNA right border primer#1
	TR2	5′-AgATgCCTCTgCCgACAgTg-3′	T-DNA right border primer#2
	TR3	5′-TCgTggAAAAAgAAgACgTTC-3′	T-DNA right border primer#3
	AD2-4	5′-NgACgA(g/C)(A/T)gANA(A/T)CAA-3′	Degenerate primer
	rel1-72833	5′-CgAgATAATgCAggAgTACAA-3′	SP-Primer
	rel1-39208	5′-ATTAATCggCAATAggTCTC-3′	SP-Primer
	TDNA7777	5′-TCgTggAAAAAgAAgACgTTC-3′	SP-Primer of T-DNA
	TDNA10622	5′-gCCACAACCTgAACCCgAAgAA-3′	SP-Primer of T-DNA
Antisense for *REL1*	*REL1-anti-f*	5'-ggCCgAgCTCCggTgCATgATgTACgTgAT-3'	P1301Ubinos
	REL1-anti-r	5'-ggCCggTACCgCACgCAAgAAACAggTCTA-3'	
Over-expression of *REL1*	*REL1-OE-f*	5'-ggCCTCTAgAATgAgCAggAACAACggCAAg-3'	P1301Ubinos
	REL1-OE-r	5'-ggCCgTCgACTCACCggCgTTggCgCggg-3'	
Promoter of *REL1*	*REL1pro-f*	5'-ggCCAAgCTTCACAAgACCCATATAAgAgCAggCgATT-3′	p1300GUSnos
	REL1pro-r	5'-ggCCgTCgACCCTCgCCgCCTCCACCAACACCACCTCCgA-3′	
cGFP fusion REL1	*REL1-Cgfp-f*	5'-CggAATTCCg TCATgAgCAggAACAACggC-3'	P35s-MCS-T35s
	REL1-Cgfp-r	5'-ggggTACCCC TCACCggCgTTggCgCgggC-3'	
Y2H for REL1-interacting proteins	*y2h_REL1-f*	5'-ggCCggATCCATgAgCAggAACAACggCAAg-3'	pGBKT7
	y2h_REL1-r	5'-AAAACTgCAgTCACCggCgTTggCgCggg-3'	pGBKT7
	y2h_RIP1-f	5'-CCggAATTCATgCAggAgTTCCACCCCgT-3'	LOC_Os06g17410, pGADT7
	y2h_RIP1-r	5'-CgCggATCCCTACggCAggTAgATCgTgCC-3'	LOC_Os06g17410, pGADT7
	y2h_RIP2-f	5'-CCggAATTCATgAgCTgTgAgTTCTTCCTCCCT-3'	LOC_Os10g30850, pGADT7
	y2h_RIP2-r	5'-CgCggATCCTCACTTgATACggTACTCgTAAATg-3'	LOC_Os10g30850, pGADT7
	y2h_RIP3-f	5'-CCggAATTCATggCCgTCTCTgTCCgTTg-3'	LOC_Os03g04020, pGADT7
	y2h_RIP3-r	5'-CgCggATCCCTACTTCCACTCgTgCgTgTCg-3'	LOC_Os03g04020, pGADT7

	y2h_RIP4-f	5'-CCggAATTCATggCAAAgCATggCgTTg-3’	LOC_Os01g71340, pGADT7
	y2h_RIP4-r	5'-CgCggATCCTTAgAAATTgATggAgTATgCCggC-3’	LOC_Os01g71340, pGADT7
	y2h_RIP5-f	5'-CCggAATTCATgACggTggAggTgACgTggg-3’	LOC_Os06g37150, pGADT7
	y2h_RIP5-r	5'-CgCggATCCCTATAgCCTggCgACgAggCC-3’	LOC_Os06g37150, pGADT7
	y2h_RIP6-f	5'-CCggAATTCATggCgTCTCCggCCA-3’	LOC_Os12g43380, pGADT7
	y2h_RIP6-r	5'-CgCggATCCTTATgggCAgAAgACgACTTggTAg-3’	LOC_Os12g43380, pGADT7
	y2h_RIP7-f	5'-CCggAATTCATggCATTCAgTAgCAAAgCTTg-3’	LOC_Os03g16950, pGADT7
	y2h_RIP7-r	5'-CgCggATCCTTAgCggTgCACAACgATCTTg-3’	LOC_Os03g16950, pGADT7
qRT-PCR	*Os01g64380-f*	5'-CgTCgCCgTCgTAgCTTTAA-3’	LOC_Os01g64380
	Os01g64380-r	5'-ACTgAAACgCgAAATTATTACCTAgAC-3’	LOC_Os01g64380
	OS01g64390-f	5'-ACgAgACgACgggTAATCCA-3’	LOC_OS01g64390
	OS01g64390-r	5'-ACTTCCTCTgTCgCgTgAA-3’	LOC_OS01g64390
	REL1-f1	5'-CAACggCAAgAgTTCCAAgCT-3’	LOC_Os01g64380
	REL1-r1	5'-CTCCgACgACACgCACgA-3’	LOC_Os01g64380
	REL1-f2	5'-CCACCAACACgTCgAACTgC-3’	LOC_Os01g64380
	REL1-r2	5'-TggAACTCTTgCCgTTgT-3’	LOC_Os01g64380
	actin-f	5'-AgCAACTgggATgATATggA-3’	X16280
	actin-r	5'-CAgggCgATgTAggAAAgC-3’	X16280
	DLT-f	5'-TgCggATACTCAACgCCATCA-3’	LOC_Os06g03710
	DLT-r	5'-ACTCgCCgACTCCggTgATC-3’	LOC_Os06g03710
	D2-f	5'-AgCTgCCTggCACTAggCTCTACAgATCAC-3’	LOC_Os01g10040
	D2-r	5'-ATgTTgTCggAgATgAgCTCgTCggTgAgC-3’	LOC_Os01g10040
	D11-f	5'-AgTgAAgAgggAgCATgAAggCAT-3’	LOC_Os04g39430
	D11-r	5'-ATCTgCAgggCTgAAATTgTTggg-3’	LOC_Os04g39430
	OsDWARF-f	5'-ATggTgTTggTggCgATTggggTggTTg-3’	LOC_Os03g40540

	OsDWARF-r	5'-ATgTTgTTCCgCCCCAggATgTCCAgCA-3'	LOC_Os03g40540
	OsDWARF4-f	5'-TTCATggAgCAgCACATCgC-3'	LOC_Os03g12660
	OsDWARF4-r	5'-CgggTAgCTgCACTCgAACA-3'	LOC_Os03g12660
	OsBZR1-f	5'-CgTCgCCCACCTACAACCTC-3'	LOC_Os07g39220
	OsBZR1-r	5'-TCgCCCAAATCgCAgCAT-3'	LOC_Os07g39220
	IBH1-f	5'-CCgAACCCTAACCCTAgCgT-3'	LOC_Os04g56500
	IBH1-r	5'-AggCCAgCATgTgCTTCgT-3'	LOC_Os04g56500
	OsBU1-f	5'-AAgCTTTAgCTCCAgCCACC-3'	LOC_Os06g12210
	OsBU1-r	5'-CCTgggCTgTTgTgATCCAT-3'	LOC_Os06g12210
	OsBLE-f	5'-AggACggTgCTgTTCTCTTg-3'	LOC_Os05g03150
	OsBLE-r	5'-gCAACAACATACATgggTTTTCC-3'	LOC_Os05g03150
	OsBRI-f	5'-TCAgAACAACTACCTCACCggCg-3'	LOC_Os01g52050
	OsBRI-r	5'-gCCggTTgCTCgCCAAA-3'	LOC_Os01g52050
	OsRAVL-f	5'-ACCTCgATAACCCACATCCg-3'	LOC_Os04g49230
	OsRAVL-r	5'-gAAggCAgCAgACggAAgAT-3'	LOC_Os04g49230
